# Effect of Irradiated Nanocellulose on Enhancing the Functionality of Polylactic Acid-Based Composite Films for Packaging Applications

**DOI:** 10.3390/polym17141939

**Published:** 2025-07-15

**Authors:** Ilaria Improta, Mariamelia Stanzione, Elena Orlo, Fabiana Tescione, Marino Lavorgna, Xavier Coqueret, Giovanna G. Buonocore

**Affiliations:** 1 Institute of Polymers, Composites and Biomaterials—CNR, P.le E. Fermi 1, 80055 Portici, Italy; ilariaimprota@cnr.it (I.I.); elenaorlo@cnr.it (E.O.); fabiana.tescione@cnr.it (F.T.); marino.lavorgna@cnr.it (M.L.); giovannagiuliana.buonocore@cnr.it (G.G.B.); 2 Department of Environmental, Biological and Pharmaceutical Sciences and Technologies, University of Campania “Luigi Vanvitelli”, Via Vivaldi 43, 81100 Caserta, Italy; 3 Institut de Chimie Moléculaire de Reims, CNRS UMR 7312, Campus Moulin de la Housse, Université de Reims Champagne-Ardenne, BP 1039, 51687 Reims, France; xavier.coqueret@univ-reims.fr

**Keywords:** electron beam irradiation, cellulose nanocrystals, active packaging films, polylactic acid blends, surface chemistry, antioxidant properties

## Abstract

This study investigates the combined use of electron beam irradiation (EBI) and nanotechnology to develop improved food packaging films. EBI, commonly applied for sterilization, can alter polymer microstructure, while irradiated cellulose nanocrystals (CNCs) offer enhanced functionality when incorporated into biopolymer matrices. Here, CNCs were irradiated with doses up to 50 kGy, leading to the formation of carboxyl and aldehyde groups, confirmed by FTIR analysis, as a consequence of the initial formation of free radicals and peroxides that may subsist in that original form or be converted into various carbonyl groups. Flexible films were obtained by incorporating pristine and EB-irradiated CNCs in an internal mixer, using minute amounts of poly(ethylene oxide) (PEO) to facilitate the dispersion of the filler within the polymer matrix. The resulting PLA/PEO/CNC films were evaluated for their mechanical, thermal, barrier, and antioxidant properties. The results showed that structural modifications of CNCs led to significant enhancements in the performance of the composite films, including a 30% improvement in water barrier properties and a 50% increase in antioxidant activity. These findings underscore the potential of irradiated CNCs as effective additives in biopolymer-based active packaging, offering a sustainable approach to reduce dependence on synthetic preservatives and potentially extend the shelf life of food products.

## 1. Introduction

Active packaging materials interact positively with the packaged food by slowing down or inhibiting the chemical and physical mechanisms responsible for the phenomena that cause food degradation [1,2]. In recent years, both academic research and the food industry have shown a growing interest in developing flexible packaging films with specific features, such as antioxidant activity, to prevent oxidation during storage and reduce the need for chemical preservatives in direct contact with food [3].

The synergistic integration of radiation and nanotechnology represents a promising approach for developing innovative food packaging materials, aimed at enhancing food quality and safety on a global scale. This strategy focuses on understanding the relationship between process, structure, and properties to effectively tailor the final performance of the material [3,4,5,6,7].

Radiation processing is increasingly applied to manufactured products (containing or) constituted of polymers or composites. In the radiation sterilization of healthcare and food products, one of the major applications of radiation processing, the treatment is performed to reduce the level of pathogenic contaminants with a minimal impact on the structural properties of the plastic components present in the packaging and packaged items [8,9]. Conversely, in the second main domain of application, the chemical effects of irradiation are positively exploited to tailor the macromolecular structure of plastics and/or rubbers formulated in simple or complex blends. Radiation treatments are indeed known to induce competing cross-linking and scission of polymer chains, oxidation, and grafting between the components present in the blends [10]. Enhancing the thermal resistance of thermoplastics used in the pipe and cable industries is illustrative of the positive effects associated with radiation cross-linking [11].

Nowadays, an emerging and promising approach in materials science is the development of nanocomposites with improved properties obtained by irradiating the composite after blending or by applying the radiation treatment only to one of its components, matrix or filler, prior to the mixing operation. From the standpoint of mechanical performances, introducing radiation-treated organic nanofillers can improve the dispersibility of the self-aggregated particles by modifying their surface properties as a consequence of main chain scission, oxidation, and/or grafting [12].

On the other hand, this technique can offer the advantage of generating compounds with antioxidant properties, capable of scavenging free radicals and interrupting the chain reactions responsible for rancidity and food spoilage, thereby enhancing food preservation [13,14,15].

Polylactic acid (PLA) is widely recognized for its excellent biocompatibility and biodegradability; however, its brittleness, limited thermal stability, and relatively high production costs constrain its broader application. To address these limitations, the incorporation of biodegradable and renewable fillers, such as cellulose and its derivatives, has been extensively explored as a strategy to enhance the mechanical and thermal properties of PLA while maintaining a favorable balance between performance and cost [16].

Among natural polymer-based particles, cellulose nanocrystals (CNCs) stand out as highly promising candidates for composite development, owing to their unique physical and chemical properties, natural abundance, renewability, and environmental sustainability [17,18]. CNCs also offer high stiffness, low density, ease of processing, compatibility with conventional manufacturing techniques, and cost-effectiveness [19]. Consequently, modified CNCs are increasingly used as reinforcing agents in a variety of polymer matrices—including starch, polyvinyl alcohol, and PLA—not only due to their surface functionality but also their proven ability to enhance mechanical strength and barrier performance in packaging applications [13,18,20].

Radiation processing is also widely used in the pretreatment of cellulosic materials, offering rapid processing and eliminating the need for chemical reagents. It has been applied in the production of modified cellulose derived from sources such as cotton linters and softwood fibers for the extraction of CNCs [21]. Aqueous suspensions of CNCs have been introduced in radiation curable polyurethane acrylate emulsions with larger enhancement of the mechanical properties after electron beam (EB) treatment than for conventional UV curing [22]. Radiation-mediated grafting of synthetic polymers with lower critical solution temperature in water has been successfully achieved to impart CNCs with thermally switchable surface properties [23]. The physicochemical properties of both pristine and irradiated CNCs have been investigated, with particular focus on the formation of new functional groups, thermal stability, and hydrophilicity [24]. These studies have shown that increasing the dose promotes the development of new chemical functionalities, thereby enhancing the antioxidant capacity of CNCs [13]. In a related study, the same research group examined the synergistic effect of irradiation and gallic acid, demonstrating improved antiradical activity in antioxidant-enriched, gellan-based film packaging designed to extend the shelf life of pre-packaged foods [25].

In this context, our aim was to prepare and assess innovative packaging film material based on PLA and irradiated cellulose nanocrystals (CNCs) with the dual objective of enhancing its overall performance and imparting antioxidant functionality. The degree of dispersion of the nanoparticle additive plays a key role in the beneficial results expected for the nanocomposites compared to the original matrix material. For the sake of simplicity in the practical implementation of this approach, we have decided to examine, at this stage, the properties of blends prepared by incorporating pristine and EB-irradiated CNCs, as obtained, in an internal mixer, using minute amounts of poly(ethylene oxide) (PEO) to facilitate the dispersion of the filler within the polymer matrix.

## 2. Materials and Methods

### 2.1. The Materials and Chemicals

Cellulose nanocrystals (CNCs, Ref. NG01NC0101) were obtained from Nanografi (Jena, Germany), with a cross-section of 10–20 nm, and they exhibited a degree of crystallinity of 92% (X-ray diffractometry).

Polylactic acid polymer (Ingeo™ Biopolymer 4032D Mw = 180,000–260,000 g/mol, with specific gravity 1.24, MFR 7 g/10 min at 210 °C/2.16 kg, density 1.08 g/cm^3^ at 230 °C, melting point 155–170 °C) was supplied by NatureWorks™ (Minnetonka, MN, USA). Prior to further processing, the PLA pellets were dried at 90 °C for at least 12 h under a vacuum due to their tendency to hydrolyze.

Poly (ethylene oxide) (PEO) (Sigma-Aldrich^®^, Milan, Italy), average Mv of ~4,000,000 g/mol (nominal), containing <1000 ppm BHT as an inhibitor, viscosity of 1650–5500 cP, 1% in H_2_O (25 °C, Brookfield) (lit.), and density of 1.21 g/mL at 25 °C, was added as a plasticizer.

### 2.2. Irradiation Procedure

The solid samples equilibrated in air containing 55% relative humidity at 23 °C were placed into sealable plastic bags before irradiation. Electron beam (EB) irradiations were performed at Ionisos (Chaumesnil, France) using a 10 MeV, 33 kW Linac accelerator at a time-averaged dose rate of approximately 4 kGy s^−1^. Samples were treated to achieve the desired doses of 25 or 50 kGy, within the range suggested by [13], by applying one or two 25 kGy passes to limit undesirable effects due to the rise in temperature during irradiation. The obtained powders were coded as CNC_25_ and CNC_50_, respectively.

### 2.3. PLA-Based Film Preparation

PLA-based films containing two different amounts (1% and 2% wt/wt) of both pristine and EB-irradiated CNC were prepared by a two-step melt process involving melt mixing and hot compression. To improve the dispersion of CNC within the polymer matrix, PEO, used as a plasticizer, was added to the formulation in weight amount equal to that of the CNC [26,27]. The selected PLA grade provides a good balance between structural integrity and thermal stability, while the ultra-high-molecular-weight PEO enhances nanoparticle dispersion and serves as a compatibilizer. This combination has been shown to effectively balance mechanical performance and processability in CNC-reinforced systems [26,27], and it was therefore chosen to promote homogeneous filler distribution and ensure the functional performance of the resulting films.

In detail, different concentrations of PEO and CNC were mixed with PLA in an internal mixer (Rheomix^®^ 600 Haake, Vreden, Germany) with a volume capacity of 50 cm^3^, at 180 °C and at 50 rpm, for 3 min. Composite films were prepared by hot pressing the obtained melt using a Collin P300P (COLLIN Lab & Pilot Solutions GmbH, Maitenbeth, Germany) press at the same previous temperature value (P = 50 bar for 3 min).

**Figure 1:**
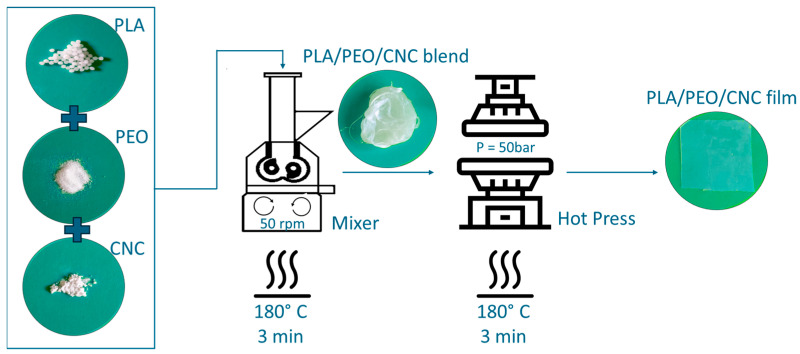
Schematic representation of the preparation process for PLA/PEO/CNC composite films.

A schematic representation of the preparation process is provided in Figure 1.

Six different film composites were obtained with a thickness of approximately 100–130 μm, whose formulation and codes are reported in Table 1. For comparison, PLA/1PEO and PLA/2PEO films without CNC fillers were prepared under the same processing conditions.

### 2.4. Experimental Characterization of CNC Powder and PLA-Based Films

#### 2.4.1. Scanning Electron Microscope (SEM)

To assess the dispersion of both pure and irradiated CNC powders within the polymer matrix, a morphological analysis was performed by using a scanning electron microscope (Quanta 200 FEG, FEI, Eindhoven, The Netherlands). Prior to analysis, dried specimens were mounted on aluminum stubs, using carbon adhesive discs, and coated with a thin layer (~10 nm) of an Au-Pd alloy, using a sputter-coating system (Emitech K575, Quorum Technologies LTD, Ashford, UK). A size distribution analysis of CNC aggregates was carried out using ImageJ software (version 1.53k). A total of 40 aggregate diameters were manually measured from SEM micrographs to construct the size distribution.

#### 2.4.2. Wide-Angle X-Ray Scattering (WAXS)

The structural characterization of CNCs was studied by WAXS, using an Anton Paar SAXS camera (Graz, Austria) equipped with a 2D imaging plate detector. CuKa X-rays were generated by a Philips PW3830 (Malvern, Worcestershire, UK), with a wavelength of 1.5418 Å, sealed tube source (40 kV, 50 mA), and slit collimation. To obtain the diffractogram, the CNC powder was analyzed for 10 min. Using an empirical method, known as the Segal or peak height method [28,29], the crystallinity index (CI, %) was determined as follows:

(1) $$CI=\frac{I_{200}-I_{am}}{I_{200}}\times \;100\%$$

where *CI* is the relative degree of crystallinity, *I*_200_ is the maximum intensity of the crystalline peak corresponding to 2θ ≈ 22.5° (in arbitrary units), and *I_am_* is the diffraction intensity of the amorphous halo (in the same arbitrary units) at 2θ ≈ 18°.

#### 2.4.3. FTIR Analysis

FTIR spectra of CNC powders and PLA-based films were collected at room temperature by using a Frontier FTIR/NIR spectrometer (PerkinElmer, Rodgau, Germany) in the attenuated total reflectance (ATR) mode from 650 to 4000 cm^−1^. Spectra were recorded at 4 cm^−1^ resolutions, and the reported results are the average of 64 scans. To better inspect the spectrum of the irradiated CNC powder and to evaluate the CNC changes before and after irradiation, the spectral region was deconvolved using Origin-Pro 8.0 and analyzed between 1500 and 1800 cm^−1^, using the Lorentzian function to fit the absorption peak identified by visual inspection of the spectra. The band positions, corresponding to specific vibrational mode assignments of the C = O stretching mode, were determined by the automatic peak-finding function [30,31].

#### 2.4.4. Analysis Thermogravimetric Analysis (TGA)

The thermal stability of the film samples was determined by using a Q500 thermogravimetric analyzer (TA Instruments, New Castle, DE, USA). Each CNC powder sample was placed in platinum trays, and it was heated through the temperature range up to 800 °C, at a rate of 10 °C/min, under nitrogen atmosphere. Similarly, each film sample of approximately 8 mg weight and of 0.5 mm diameter was placed in platinum trays and heated up to 600 °C at a rate of 10 °C/min, under nitrogen atmosphere.

#### 2.4.5. Differential Scanning Calorimetry (DSC)

All tests were performed on a Q2000 Differential Scanning Calorimeter (DSC) (TA Instruments, New Castle, DE, USA) in a heat–cool–heat mode and processed with the TA Universal Analysis software (ver. 4.1 D). Each sample (3–5 mg) was placed in an aluminum DSC pan and heated from –80 °C to 250 °C. The acquired data were processed by the TA Universal Analysis software to calculate all the parameters, and the degree of crystallinity was calculated from the corresponding melting enthalpy values of the second scan according to the following relationship:

(2) $$x_{c}=\frac{{∆H}_{m\;}-{∆H}_{cold\;}\;}{{∆H}_{m100\;}\cdot \;W_{f\;}}\;\cdot 100$$

where Δ*H_m_*, Δ*H_cold_*, and Δ*H_m_*_100_ are the enthalpies of melting, cold crystallization, and 100% crystalline PLA, respectively, taken as 93 J/g, while *W_f_* is the weight percent of PLA for the specific sample [32].

#### 2.4.6. Mechanical Properties Tests

Mechanical behavior was assessed in tensile configuration, using a Lonost T1 Universal Testing Machine (Lonos srl, Monda, Italy), equipped with a 250 N load cell and set at a crosshead speed of 5 mm/min in accordance with ASTM D882 [33]. Data from 10 different tests were averaged for statistical purposes.

#### 2.4.7. Barrier Properties Tests

Water vapor permeability was determined on 5 cm^2^ samples, using infrared sensor technology with a PermatranW3/31 (Mocon GmbH, Neuwied, Germany) at 25 °C. Permeation tests were performed with relative humidity set at 0% and up to 50% on the downstream and upstream sides of the film, respectively, under a nitrogen stream flow rate of 100 mL/min. Each test was performed in duplicate.

#### 2.4.8. Surface Wettability: Contact Angle Measurements

All the measurements were carried out using the Dataphysics OCA 20-Optical (Filderstadt, Germany) contact angle instrument, dispensing a 1 mL drop of water to assess the wettability of the surface or of diiodomethane to assess the improvement in printability, i.e., ink adhesion on the surface. The solvent was dropped on at least 10 different sites of the tested samples, and the static contact angle was reported as the average value of each individual measurement.

The surface free energy ($\gamma_{s}$) of the films was calculated using the Owens–Wendt method, which separates $\gamma_{s}$ into dispersive ($\gamma_{s}^{d}$) and polar ($\gamma_{s}^{p}$) components [34]. The method uses contact angle measurements (θ) with two standard liquids whose surface tension values ($\gamma^{L}$) and components are known:

Water: $\gamma^{L}$ = 72.8 mN/m ($\gamma^{Ld\;}$= 21.8, $\gamma^{Lp}\;$= 51.0).

Diiodomethane: $\gamma^{L}$ = 50.8 mN/m ($\gamma^{Ld}$ = 50.8, $\gamma^{Lp}$ = 0.0).

The following equation was applied:

(3) $$\gamma^{L}(1+cos\theta )=2[\sqrt{\gamma_{s}^{d}{\cdot \gamma }^{Ld}}+\sqrt{\gamma_{s}^{p}{\cdot \gamma }^{Lp}}]$$

Solving the system for both liquids yielded the polar and dispersive components of $\gamma_{s}$. The total surface energy was calculated as $\gamma_{s}$ = $\gamma_{s}^{d}$+ $\gamma_{s}^{p}$.

#### 2.4.9. Color Analysis Measurements

The color analysis of the films was carried out by using a colorimeter (Minolta Chroma Meter, CR 300, Osaka, Japan). Hunter parameters are L* (from 0 = black to 100 = white), a* (−a* = green to + a* = red), and b* (−b* = blue to + b* = yellow). For each film, three random measurements in different positions were taken and averaged. The results are expressed as the difference between the color parameters of the sample and the color parameters of a reference standard (L = 96.3, a = +0.5, b = +2.9) used as the background. The total color difference (Δ*E*) was then calculated using the following equation [35]:

(4) $$∆E=\sqrt{{\left(L_{film}-L_{standard}\right)}^{2}+{\left(a_{film}-a_{standard}\right)}^{2}+{\left(b_{film}-b_{standard}\right)}^{2}}$$

#### 2.4.10. ABTS Radical Scavenging

The antiradical activity of PLA films with and without PEO and CNC was tested using the ABTS assay according to the procedure already reported in previous works [36,37]. In detail, aqueous solutions of ABTS (7 mM) and K_2_S_2_O_8_ (140 mM) were co-incubated for 16–18 h in the dark to obtain the ABTS•+ solution. Then, 227 µL of ABTS•+ solution (0.7 ± 0.02 OD at 734 nm) was placed in contact with 0.19 cm^2^ of PLA films in quadruplicate, using 96-well microtiter plates. After 6 min, the films were removed, and the absorbance at 734 nm was recorded. The radical scavenging percentage (RS%) was calculated using the following formula:

(5) $$RS\%=\left[\frac{{OD}_{negative\;control}-{OD}_{sample}}{{OD}_{negative\;control}}\right]$$

## 3. Results

### 3.1. Powder Characterization

#### 3.1.1. Morphological Powder Analyses

**Figure 2:**
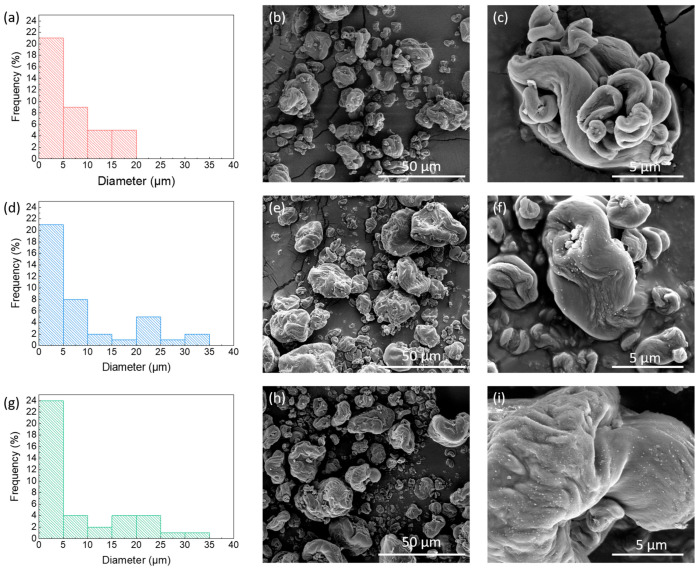
SEM analysis with magnification 2500X and 20,000X for (**b**,**c**) CNC pristine, (**e**,**f**) CNC_25_, and (**h**,**i**) CNC_50_; and size distribution of (**a**) CNC pristine, (**d**) CNC_25_, and (**g**) CNC_50_.

SEM analysis was carried out to investigate the effect of irradiation on the morphology of the three different powders. The results reveal that the pristine CNC (Figure 2b) has irregularly shaped aggregates, which become smaller and more irregular as the irradiation dose increases (Figure 2e,h). At 20,000X magnification, the aggregates appear with a smooth surface (Figure 2c), while both CNC_25_ and CNC_50_ (Figure 2f,i) exhibit small structures on the surface that increase in number at higher doses.

The histograms on the left in Figure 2a,d,g show the diameter distribution of the CNC aggregates in each sample. As the irradiation dose increases, a clear shift towards larger aggregate sizes is observed. The CNC sample shows a narrower size range, indicating greater homogeneity. Increasing the radiation dose results in a population of smaller diameters; however, gradually larger aggregates also appear.

**Figure 3:**
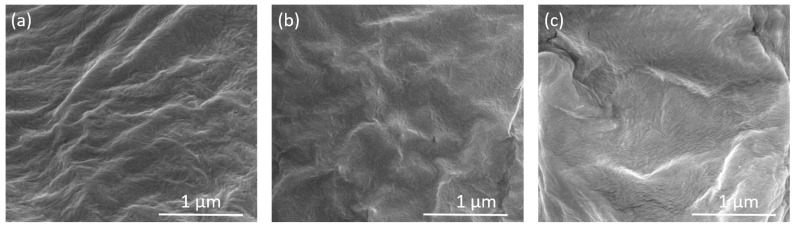
SEM micrographs recorded at the same magnification for CNC aggregates: (**a**) pristine CNC, (**b**) CNC_25_, and (**c**) CNC_50_.

At 100,000X magnification of the aggregate surface (Figure 3), it is evident that the population density of CNC chains rises with the increasing radiation dose, and they also arrange into a compact and continuous morphology at higher doses.

Irradiation induces the formation of macroradicals, which subsequently react with one another to create cross-links between polymer chains. This process reduces chain mobility and alters the submicroscopic capillarity of the cellulose fiber structure. Morphologically, these effects are evidenced by the formation of larger aggregates, visible at higher SEM magnifications, as well as by surface regions where individual layers of cellulose chains are no longer distinguishable. This indicates a more compact morphology [38].

**Figure 4:**
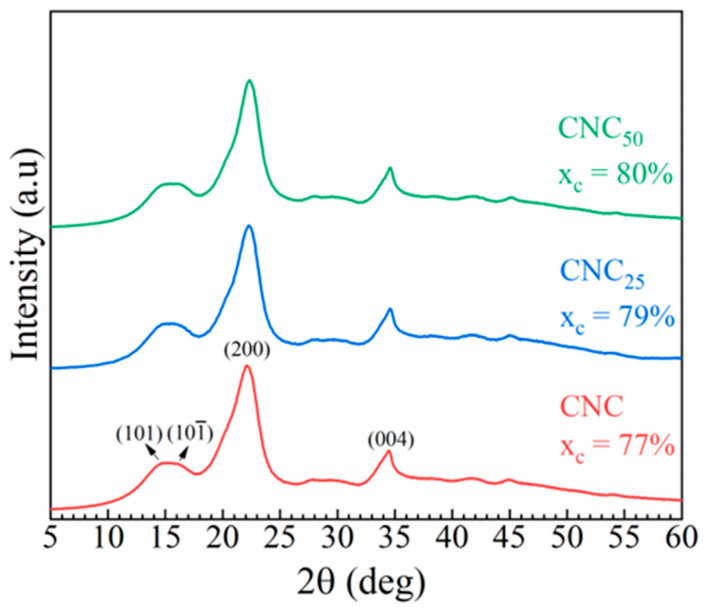
WAXS spectra of pristine CNC (red line) and CNC_25_ (blue line) and 50 kGy (green line).

WAXS analysis was performed to determine the crystallinity of CNCs (Figure 4). The diffractogram of the unirradiated CNCs exhibits a typical Form I cellulose pattern, with two main peaks at 2θ around 15.3°, corresponding to the overlap of reflection planes (101) and (101), and at 2θ equal to 22.1°, associated with the reflection plane (200) [28]. The diffraction patterns of the irradiated CNCs confirm that cellulose crystallinity remains in Form I when irradiation is performed on the solid and moisture-free materials. Cellulose can adopt several crystalline polymorphs—namely cellulose I, II, III, and IV—depending on its botanical origin and posttreatment. Form I, the native crystalline structure of plant-derived cellulose, consists of two allomorphs: Iα, predominantly found in algae and bacteria, and Iβ, which is prevalent in higher plants. This form is characterized by a parallel chain arrangement and distinct sharp diffraction peaks in WAXS patterns. It is typically retained in cellulose nanocrystals (CNCs) obtained via acid hydrolysis [39].

As the irradiation dose increases, the degree of crystallinity rises from 77% in the unirradiated CNC to 80% in the CNC_50_. This is a general feature of natural and synthetic semicrystalline polymers in which chain scission induced by irradiation at low dose allows some segments of tie-chains to gain more mobility and to develop ordered interactions with the neighboring crystals. This common phenomenon increases the fraction of crystalline domains within the material [40,41].

**Figure 5:**
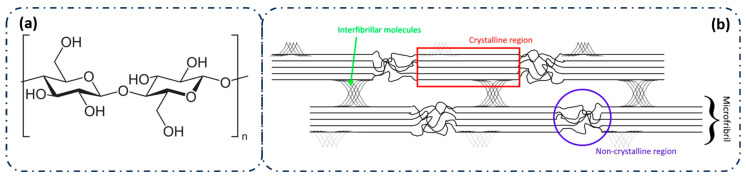
(**a**) Chemical structure of the cellulose repeating unit within the polymer structure. (**b**) Simplified structure of cellulose microfibrils, showing the presence of amorphous and crystalline zones. Adapted from Sabara et al. [42], licensed under CC BY 4.0.

Figure 5a,b provides a schematic representation of the molecular and structural phases of CNC. Pristine CNC is characterized by parallel chains linked by strong hydrogen bonds, forming microfibrils. Figure 5b illustrates the structure of cellulose microfibrils, which are locally ordered with both amorphous and crystalline regions, exhibiting different crystal structures [26,35].

**Figure 6:**
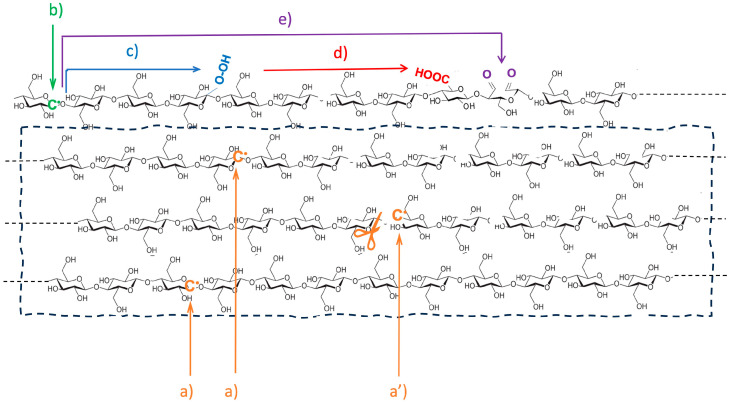
Sketch representing the main chemical events taking place in irradiated cellulose nanocrystals: radiolytic formation of C-centered long-lived free radicals in the core of nanocrystals (a), with only a few of them undergoing chain scission (a’); radiolytic formation of C-centered free radicals in chains at the surface of nanocrystals with more mobility and in contact with gaseous environment and moisture (b), further converted into peroxyl radicals and then peroxides by reaction with molecular oxygen (c), and finally leading to carbonyl derivatives (d), with some carbonyl derivatives being directly formed by multistep evolution of the initial radical formed on an anhydroglucose unit (e).

From a chemical standpoint, irradiation of CNCs in the presence of air at doses of 25 and 50 kGy can be summarized as follows. High energy carried by the penetrating fast electrons is deposited in the irradiated matter without selectivity. The primary ionization and excitation processes take place within the core of crystalline domains, in the less ordered chains at the surface and in the remaining tie chains linking the nanocrystals. Since the results of XRD analyses establish that the crystalline structure is not degraded and the crystallinity degree is even slightly enhanced, the chemical transformations following primary stages would essentially take place in the less ordered domains surrounding the almost perfectly packed chains. If we neglect the indirect effects of water radiolysis that may generate oxidizing HO^•^ radicals in the slightly hydrated surface layer of CNCs, C-H bond dissociation is expected to yield C-centered free radicals at the C(1) or C(4) position of anhydroglucose units [43]. A simplified description of the other events taking place in the CNCs is illustrated in Figure 6.

In crystalline domains, radiolysis induces the formation of long-lived C-centered free radicals on anhydroglucose units of the cellulose chain together with H atoms that can recombine to form gaseous hydrogen. Scission reactions within the polysaccharide chains cannot be excluded but seem very limited even at 50 kGy, as a probable consequence of the high order and minimal mobility in the purified nanocrystals.

At the surface of CNCs, the C-centered free radicals (label b) can undergo main chain scission, but in the presence of air, the radicals would readily react with molecular oxygen to produce hydroperoxides (reaction c) or peroxides, some of which are further converted into carbonyl derivatives (reaction d). Alternatively, there is a cascade of reactions and rearrangements involving free radical species located on close or open anhydroglucose units [44]. The decomposition of peroxides is subject to thermal activation during storage, blending, or processing. This may gradually convert the less stable chemical entities or functions, in particular, free radicals and peroxides, while the aldehydes formed in situ would be sensitive to further oxidation into carboxylic derivatives [45]. It was one of the main motivations of this study to evaluate the beneficial consequences of the surface functionalization of CNCs on the mechanical and functional properties of the nanocomposite thermoplastic films containing irradiated CNCs. Moreover, the presence of reactive functions was expected to favor development at different stages, particularly in the subsequent blending and film production operated at elevated temperatures. In such conditions, the decomposition of peroxides and possible hopping process transferring the free radical species at the surface of CNCs would induce coupling reactions between the nanofiller and the thermoplastic matrix, with some enhancement of materials properties.

#### 3.1.2. Structural Powder Analyses

**Figure 7:**
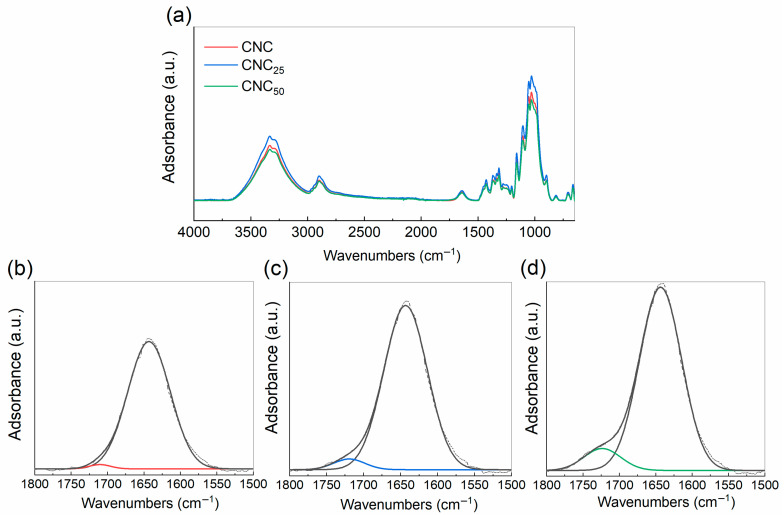
FTIR (**a**) of untreated CNC (black line) and CNC_25_ (red line) and 50 kGy (blue line) and deconvoluted range of C–O–C vibration bands related to data of untreated CNC (**b**), and CNC_25_ (**c**) and CNC_50_ (**d**) (1500–1800 cm^–1^). Within the (**a**–**c**) deconvoluted range, black dashed lines represent the acquired FTIR signals.

The FTIR spectra of unirradiated and irradiated CNCs are shown in Figure 7, revealing structural changes in the irradiated CNCs. All spectra exhibit characteristic peaks associated with the CNC chemical structure. Specifically, the large band at 3340 cm^−1^ corresponds to the -OH stretching vibration, while the peaks at 2890 cm^−1^ and 1431 cm^−1^ are indicative of C-H stretching and -CH_2_ group bending, respectively. The peaks at 1160 cm^−1^ and 1070 cm^−1^ are attributed to the saccharide structure (Figure 7a) [19]. Notably, the intensity of the peak at 1733 cm^−1^, corresponding to the C=O stretching mode [46], increases with the irradiation dose, a trend that is most clearly observed in the deconvolved plots of Figure 7c,d. Along with the formation of carbonyl groups, the peak at 1642 cm^−1^ [47,48], related to the amount of adsorbed water, changes and increases with the applied doses from 25 to 50 kGy. This phenomenon is likely linked to the formation of new hydrophilic groups, such as carboxylic acid groups, due to the cleavage of glycosidic bond [13]. The cleavage of cellulose chains could affect the crystalline rearrangement, leading to a slight increase in the crystallinity (as indicated by the WAXS results) due to shorter and more easily crystallized chains.

**Figure 8:**
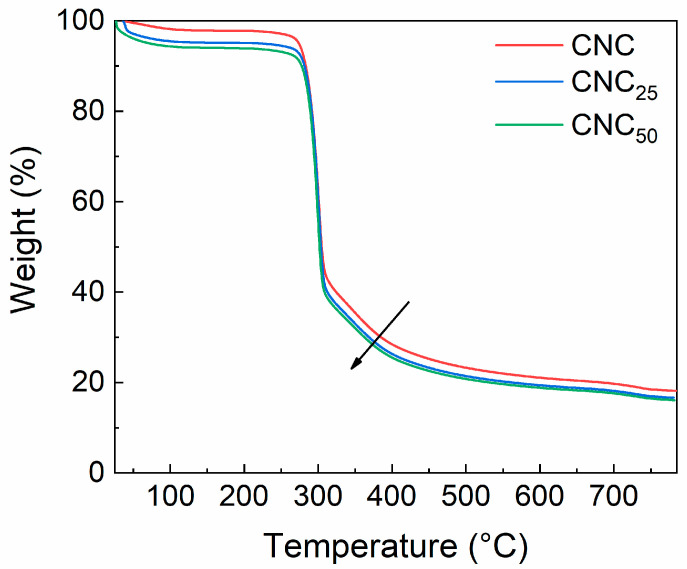
TGA data of CNC (red line), CNC_25_ (blue line), and CNC_50_ (green line). The arrow represents the thermal stability trend of unirradiated and irradiated CNCs.

The thermal decomposition behavior of both unirradiated and irradiated CNCs, as well as the weight loss as a function of temperature, is presented in Figure 8. The thermograms display four distinct regions: (1) water loss (below 100 °C), (2) depolymerization (around 250 °C), (3) thermal decomposition of cellulose (around 310 °C), and (4) residual char (above 400 °C). The weight loss starts at below 100 °C, and this is due to the evaporation of adsorbed bulk water [14]. Indeed, the TGA curves for CNC_25_ and CNC_50_ powders (represented by the blue and green curves, respectively) show a decrease in thermal stability as the radiation dose increases, as also reported in the literature [49]. This behavior is attributed to the formation of oxidized groups, such as carboxyl and aldehyde functionalities, as well as the breakdown of glycosidic bonds during electron beam irradiation. These structural changes lead to a reduction in molecular weight and an increased presence of thermally labile species, which promote an earlier onset of thermal degradation. Reduced thermal stability is observed after a weight loss of approximately 60% of the initial powder. The shift in weight loss to lower temperatures is typical of oxidized cellulose and occurs between 300 and 400 °C. Weight loss at 350 °C was 64.6%, 67.1%, and 68% for CNC, CNC_25_, and CNC_50_, respectively. The residual mass at 800 °C was 83.64%, 83.32%, and 84.19% for CNC, CNC_25_, and CNC_50_, respectively.

### 3.2. Film Characterization

#### 3.2.1. Morphological Analyses of Films

**Figure 9:**
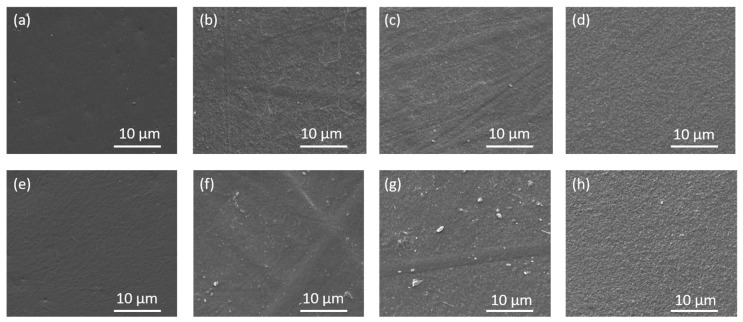
SEM analysis of pristine (**a**) PLA/1PEO, (**b**) PLA/1PEO/1CNC (**c**) PLA/1PEO/1CNC_25_, (**d**) PLA/1PEO/1CNC_50_, (**e**) PLA/2PEO, (**f**) PLA/2PEO/2CNC, (**g**) PLA/2PEO/2CNC_25_, and (**h**) PLA/2PEO/2CNC_50_.

Morphological analysis of pure PLA films and nanocomposites with varying percentages of CNC was conducted to evaluate changes in the film surface. Figure 9 presents SEM micrographs of different types of films loaded with unirradiated and irradiated CNC at two different doses. In the analysis of the PLA/PEO film (Figure 9a,e), it is observed that as the percentage of PEO in the matrix increases, the population density of the polymer chain structure also increases. Consequently, when pure CNC powder is added to the PLA/2PEO/2CNC film, the CNC chains are incorporated into the matrix structure. In contrast, in the PLA/1PEO/1CNC film, the CNC chains are more free and therefore more visible on the surface of the sample. CNC_25_, having a structure with more free radicals, interacts more with the polymer matrix when added, altering its structure. In the PLA/1PEO/1CNC_25_ film, small stretches of the CNC chain remain visible on the surface, whereas in the PLA/1PEO/1CNC_50_ film, they are completely absorbed by the matrix, creating a more uniform and smoother surface. In the PLA/2PEO/2CNC_25_ films, fibrils and agglomerates appear in some areas of the surface. In the PLA/2PEO/2CNC_50_ films, the surface is homogeneous, but with a denser structure compared to the PLA/1PEO/1CNC_50_ film.

#### 3.2.2. Structural Analyses of Films

FTIR analysis was performed to investigate possible chemical interactions between the PLA backbone, PEO, and both pristine and irradiated CNCs. The dominant interaction mechanisms are not fully understood and likely depend on the specific surface chemistry of the CNCs; however, all PLA-based composites exhibit the characteristic infrared peaks of the PLA matrix, and no significant changes in vibrational shifts were observed. Specifically, the absorption peaks at 2997 and 2945 cm^−1^ correspond to the –CH_2_ asymmetric and symmetric vibrations, respectively. A strong absorption band at 1747 cm^−1^ was observed, which is attributed to the C=O stretching of PLA. The bending vibrations in the 1300–1500 cm^−1^ range are associated with the antisymmetric and symmetric bending of the –CH_3_ group. The bands at 1266 and 1209 cm^−1^ are attributed to the C–O–C antisymmetric and symmetric stretching in esters, while the peak at 1180 cm^−1^ is linked to the C–O–C stretching of PLA. The three peaks at 1125, 1080, and 1040 cm^−1^ may correspond to C–O stretching vibrations. The last two bands, detected at 868 and 755 cm^−1^ (in the far infrared region), can be assigned to the amorphous and crystalline phases of PLA. The values corresponding to these peaks are summarized in Table 2 [20].

**Figure 10:**
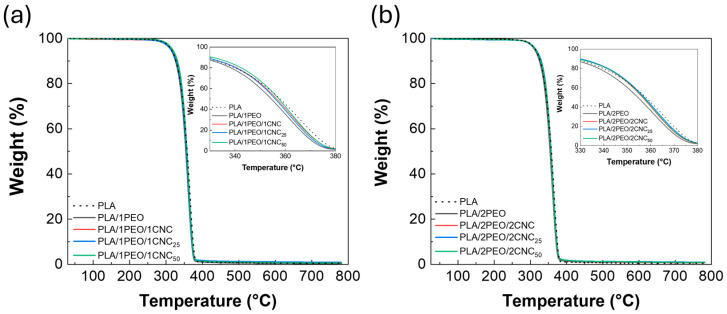
TGA data of PLA/PEO (**a**) and PLA/PEO/CNC composites (**b**).

The thermal stability, including degradation, crystallinity, and glass transition, of neat PLA and its corresponding composites, both with different amounts of PEO and unirradiated or irradiated CNC, was investigated using TGA and DSC. Figure 10 displays the thermogravimetric curves for PLA with 1% wt (a) and 2% wt (b) of both PEO and CNCs. All the curves exhibit the characteristic behavior of the PLA matrix, with a single degradation step occurring around 370 °C. The addition of PEO results in lower thermal stability for the resulting composites (i.e., PLA/1PEO and PLA/2PEO) compared to pristine PLA, due to the plasticizing effect of PEO. The incorporation of varying amounts of unirradiated and irradiated CNC helps minimize the effect of PEO, as CNC interacts with the bonds formed between PLA and PEO during crystallization, thus reducing chain mobility [50].

**Figure 11:**
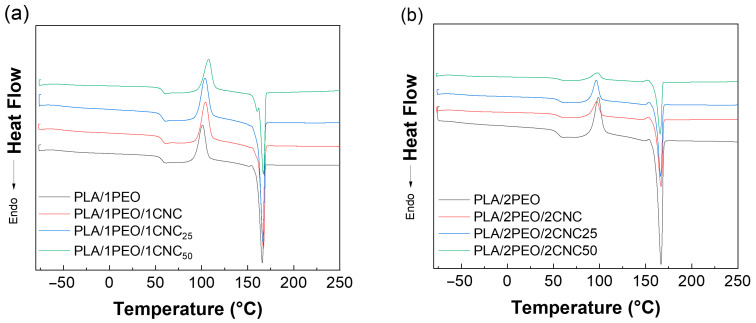
DSC data (second heating cycle) of (**a**) neat PLA/1PEO and PLA/1PEO/1CNC and (**b**) neat PLA/2PEO and PLA/2PEO/2CNC films.

The DSC thermograms shown in Figure 11 were analyzed, and the main characteristic parameters related to the second heating are reported in Table 3. In the PLA/PEO blends, the Tg decreases for both PEO amounts compared to the pristine PLA value, confirming that PEO acts as a plasticizer. However, the degree of crystallinity increases. The presence of PEO can promote the crystallization of PLA and enhance the mobility of its molecular chains, thereby accelerating the crystallization rate, allowing PLA to crystallize more extensively during cooling and at higher temperatures [50,51]. The incorporation of both unirradiated and irradiated CNCs appears to reduce the plasticizing effect of PEO. Specifically, at higher PEO levels, the crystallinity degree of the films remains constant, possibly due to a balance between the enhanced chain mobility induced by PEO and the structural influence of CNC aggregates. In contrast, at lower PEO amounts, the observed decrease in crystallinity (Xc) with higher doses of CNC irradiation may be due to the generation of trapped free radicals and surface (hydro)peroxides, which induce coupling reactions during processing. This may reduce the time available for orderly crystallization of PLA chains, leading to a more amorphous morphology [13].

#### 3.2.3. Mechanical Properties

Mechanical characterization was carried out to evaluate the effect of pristine and irradiated CNC on the behavior of nanocomposite films, in comparison to PLA/PEO films. Table 4 presents the mean values and corresponding standard deviations for Young’s modulus, stress, and elongation at break.

Incorporation of PEO serves two primary functions compared to PLA. First, it acts as a plasticizer, lowering the glass transition temperature and increasing chain mobility. While pure PLA has a crystallinity of around 9%, PLA/PEO blends exhibit significantly higher crystallinity due to better chain rearrangement during cooling, as supported by the literature [52]. Generally, higher crystallinity leads to increased stiffness and tensile strength, whereas lower crystallinity results in greater ductility and flexibility. Consequently, the mechanical behavior of PLA/PEO/CNC films is influenced by both CNC dispersion and changes in matrix crystallinity induced by the presence of PEO.

In the case of films containing 1% *w*/*w* of CNC, it is observed that the addition of pristine CNC increases Young’s Modulus by approximately 18%, acting as a reinforcement, without altering the fracture stress of the films compared to PLA/1PEO. However, the presence of CNC_25_ leads to a 33% reduction in mechanical performance, likely due to the fracture of long polymer chains. In contrast, the addition of CNC_50_ results in a 13% recovery of breaking strength and a 40% increase in film elongation, probably due to its improved homogeneity within the polymer matrix. For the PLA/1PEO/1CNC_25_ and PLA/1PEO/1CNC_50_ films, a decrease in Young’s modulus and slight elongation at break are observed, which can be attributed to chain breakage and the consequent formation of radicals [35,51].

Regarding the 2PEO:2CNC ratio, it is shown that the addition of CNC to the PLA/PEO films leads to an 8% reduction in maximum stress and modulus while maintaining the same elongation at break. This is likely due to the coarse dispersion of CNC and the non-homogeneous morphology, which induces the formation of agglomerates and voids that act as stress concentrators [19]. The improved dispersion of CNCs in PLA/PEO blends can be attributed to several complementary mechanisms. The flexible chains and low glass transition temperature (*T_g_*) of PEO facilitate CNC mobility during the melt processing, while its hydrophilic character promotes hydrogen bonding with the hydroxyl-rich surfaces of CNCs. These interactions enhance compatibility and suppress aggregation. Additionally, PEO contributes to steric stabilization and may act as a molecular bridge between PLA and CNCs, thereby improving interfacial adhesion and facilitating more efficient stress transfer across the matrix [26,27].

In the case of PLA/PEO blends containing CNC_25_, both the maximum stress and elongation increase by approximately 28% and 42%, respectively, while the modulus remains constant. This improvement can be attributed to the better dispersion of the filler within the polymer matrix, which enhances the strength of the film without compromising its flexibility. On the other hand, the use of CNC_50_ results in a 19% loss in strength and a slight 5% reduction in modulus. In this case, further irradiation of the CNC leads to an excessive increase in surface density, which negatively affects the mechanical behavior despite the more homogeneous surface. Criado et al. [25] observed enhanced mechanical performance in gellan gum films when gallic acid was grafted using pre-irradiated CNCs at various concentrations, with the best results at 20 wt%. In contrast, the present study achieved similar mechanical improvements using only 2 wt% of pre-irradiated CNCs, suggesting a more efficient reinforcing effect at substantially lower filler content.

Moreover, when comparing samples with different CNC concentrations, the results indicate that the addition of CNC irradiated at different doses provides lower stiffness and therefore higher elongation at break in both 1:1 and 2:2 ratios. This makes the PLA/PEO/CNC films more processable and flexible.

#### 3.2.4. Water Permeability and Surface Wettability

**Figure 12:**
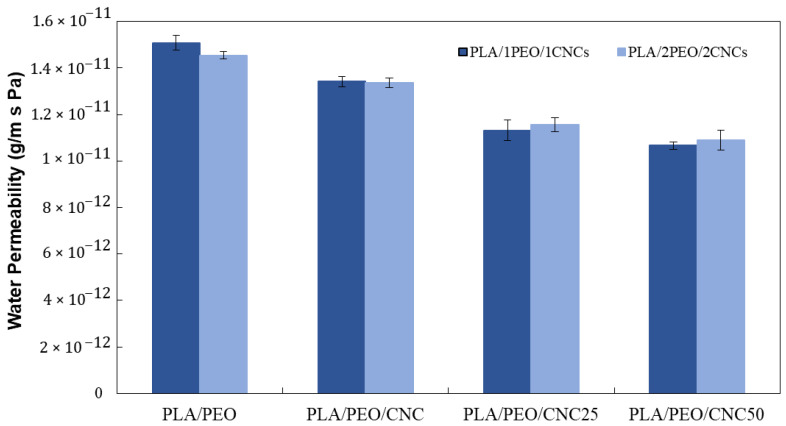
Water permeability data of PLA/PEO and of composite films containing pristine and irradiated CNC (blue bars: PLA/1PEO/1CNCs; light blue bars: PLA/2PEO/2CNCs).

The influence of unirradiated and irradiated CNCs on the barrier properties of the films was evaluated by measuring water vapor permeability (Figure 12). It can be inferred that the increase in PEO has a negligible effect on water permeability values, as does the presence of pristine CNC, which leads to only a 10% increase in the water barrier. Conversely, the presence of irradiated CNC results in a more significant decrease in water permeability, with reductions of approximately 25% and 29% for PLA/PEO/CNC_25_ and PLA/PEO/CNC_50_, respectively. A reduction in water vapor permeability was also observed by Criado et al. [25], although significant improvements were only achieved at a CNC loading of 20 wt%. In contrast, the present study attained a comparable 29% reduction in WVP using just 2 wt% of irradiated CNCs, suggesting that radiation-induced surface modifications effectively enhance barrier properties of the developed PLA-based film even at low filler concentrations.

To evaluate the surface properties of the active films, contact angle measurements were performed using two different liquids (distilled water and diiodomethane). The data presented in Table 5 show that increasing the amount of PEO does not affect the wettability of the samples. On the contrary, the presence of CNC leads to an increase in surface hydrophobicity, which becomes even more pronounced with irradiated CNC, especially in films containing higher amounts of PEO and CNC. The positive effect on the wettability of PLA/PEO-based films with cellulose is mainly due to the presence of low-polarity sulphate groups on the surface that increase the hydrophobicity of the final material [47].

Contact angle measurements performed with diiodomethane (DII) reveal that films with lower PEO content exhibit lower contact angle values, regardless of the presence of CNC. Notably, films containing CNC_25_ (in both 1:1 and 2:2 ratios) show lower DII contact angle values compared to the corresponding films with and without CNC, indicating improved wettability and enhanced printability. The lower contact angle values confirm that these films offer better ink adhesion, making them more suitable for printing applications.

Moreover, the surface free energy (γ_s_) of the PLA/PEO and PLA/PEO/CNC films was calculated, including its polar (γ_s_^p^) and dispersive (γ_s_^d^) components, using the Owens–Wendt method, based on contact angle measurements with water and diiodomethane. The obtained values are reported in Table 5 and indicate that surface energy is significantly influenced by both the irradiation dose of CNCs and the PEO content. For the PLA/1PEO system, the incorporation of CNCs led to slight variations in γ_s_, with a general decrease in the polar component (γ_s_^p^) as the irradiation dose increased. This trend suggests a reduction in the availability or surface exposure of polar –OH groups, likely due to the formation of compact aggregates or cross-linked domains that shield functional groups from the interface [53].

In the PLA/2PEO series, an initial increase in surface free energy and its components was observed for PLA/2PEO/2CNC and PLA/2PEO/2CNC25, likely due to improved interfacial compatibility and more uniform CNC distribution within the more hydrophilic matrix. However, at the highest irradiation dose (PLA/2PEO/2CNC50), both the dispersive (γ_s_^d^) and total surface energy (γ_s_) decreased, suggesting a possible loss of homogeneity and increased CNC aggregation.

These results highlight the complex interplay between CNC irradiation dose, PEO content, and the PLA matrix in determining surface morphology and interfacial properties [51,54]. While a higher PEO content initially enhances wettability and surface polarity, this stabilizing effect diminishes at higher irradiation doses due to structural changes in the CNC phase.

While FTIR analysis indicated an increased intensity in the –OH region—suggesting the formation of additional polar groups upon irradiation—the Owens–Wendt analysis revealed a slight decrease in the polar component of the surface energy. This apparent discrepancy can be attributed to the morphological evolution of CNC aggregates under irradiation: although more polar functionalities may be formed, their accessibility at the surface is likely reduced due to increased aggregate compaction or internal localization of these groups within the aggregates.

#### 3.2.5. Color Analysis

Color is an important feature for consumer acceptance of packaging, as it reflects the light within the visible spectrum [35]. Table 6 presents all the color parameters of PLA/PEO and composite films containing unirradiated and irradiated CNCs. An increase in the b* value is observed in all the tested specimens, without significantly affecting the ΔE value of the composite films. In the presence of CNC_25_, the total color value increases for both 1% and 2% PEO. This change corresponds to an increase in the blue component (b*) and a decrease in the yellow component, resulting in a cooler tone compared to films with non-irradiated CNCs. Conversely, films containing CNC_50_ show a decrease in total color variation, suggesting that light diffraction is closer to a neutral state. It is important to note that, in all cases, the ΔE value remains within an acceptable range for packaging applications. Color perception studies suggest that a ΔE below 1 is generally imperceptible to the human eye, while a ΔE between 1 and 2 is only noticeable upon close inspection [55]. Values up to 3–4 are still considered acceptable, as they fall within the limits commonly used in the packaging sector [56].

### 3.3. Evaluation of Antioxidant Activity

**Figure 13:**
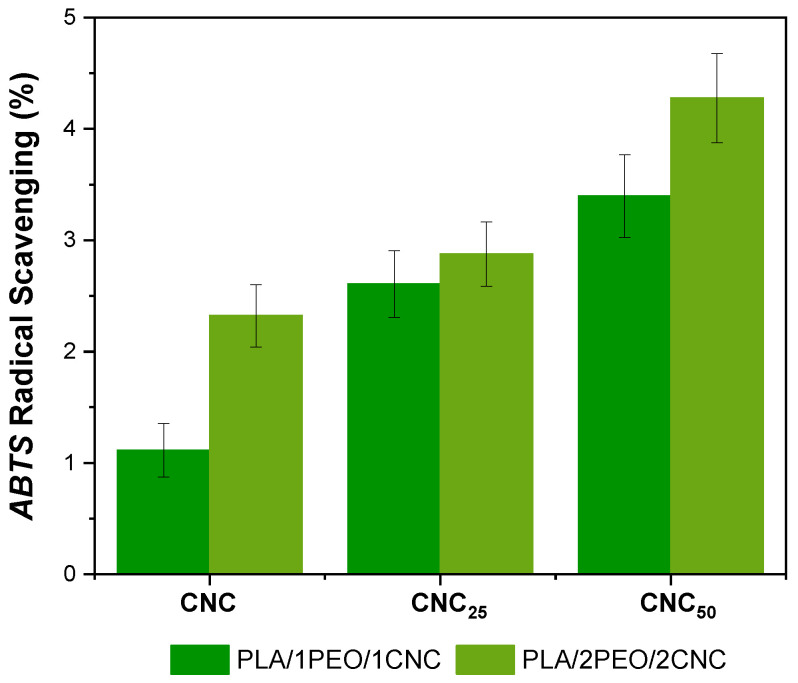
ABTS radical scavenging (%) of unirradiated and irradiated PLA films containing 1% and 2% of both PEO and CNC.

The films PLA/1PEO/1CNC and PLA/2PEO/2CNC, both in their pristine form and irradiated at different doses (25 and 50 kGy), were tested to evaluate their ability to scavenge ABTS•+ radicals. The results are presented in Figure 13.

It is evident that films containing CNC_25_ and CNC_50_ exhibit higher ABTS•+ radical scavenging percentages compared to non-irradiated samples. Interestingly, CNC irradiation proves to be an effective method to enhance the antiradical activity of these PLA-based active films. Specifically, the incorporation of CNC_25_ into PLA/1PEO/1CNC films results in an approximately 30% increase in scavenging activity, while exposure to 50 kGy leads to a 50% improvement. Figure 13 also highlights the crucial role of the irradiation dose in enhancing the antiradical properties of these materials. The highest ABTS•+ scavenging activity is observed in films containing CNC_50_. However, at a dose of 25 kGy, the improvement in antioxidant properties appears to be better balanced with the amount of CNC added, particularly in the 1:1 PLA/PEO formulation, ensuring effective radical scavenging activity, while maintaining suitable mechanical and structural properties. Criado et al. [25] reported a 49% enhancement in the antioxidant activity of gellan gum films incorporating 20 wt% CNC-g-GA. In comparison, the present study achieved a similar increase (~50%) using only 1–2 wt% of electron beam-irradiated CNCs, demonstrating a more efficient antioxidant effect at significantly lower filler concentrations in PLA-based film. The improved antioxidant behavior can be attributed to the formation of long-lived radicals and surface carbonyl groups (such as aldehydes) on the CNCs during irradiation. These species contribute to the stabilization of the polymer matrix and enhance its antioxidant activity by scavenging free radicals or interrupting oxidative chain reactions [22,23,25]. The significant reduction in color change observed in the irradiated CNC-based films (Figure 7) further supports this effect, indicating that CNCs help scavenge radicals and stabilize the polymer structure, thereby limiting the formation of chromophoric degradation products typically associated with oxidative processes [57].

## 4. Conclusions

The aim of this research was to develop an innovative active packaging film by incorporating electron beam (EB)-irradiated cellulose nanocrystals (CNCs) into a thermoplastic polylactic acid (PLA) matrix, containing poly(ethylene oxide) (PEO), acting as a compatibilizer, as a possible strategy to enhance the degree of CNC dispersion within the polymer.

The dual objectives were to enhance the final properties of the material and introduce new functionalities, such as antioxidant activity.

The study demonstrated the effect of different EB irradiation doses on the physical, chemical, mechanical, thermal, and barrier properties of PLA films containing different concentrations of both pristine CNCs and CNCs irradiated at 25 kGy and 50 kGy. Among the investigated formulations, the PLA/1PEO/1CNC film containing CNC_25_ emerged as the most promising composition.

This film showed improved mechanical properties, improved water barrier properties (consistent with water contact angle measurements); enhanced printability, as confirmed by diiodomethane contact angle measurements; and good color stability. Moreover, the modification of CNCs through EB irradiation led to the formation of new chemical functionalities, such as carboxyl and aldehyde groups, and the generation of free radicals. This effect resulted in a significant increase in antioxidant activity, making these films particularly suitable for active packaging applications. These findings confirm that the use of irradiated CNCs in packaging films represents a promising and effective strategy to improve barrier and mechanical properties, control moisture, enhance printability and consumer appeal, and impart new functionalities. All of these aspects are essential for the development of sustainable and functional packaging materials, and they are potentially usable to extend the shelf life of packaged food.

As a future prospective, it will be necessary to develop effective strategies to preserve and stabilize the antioxidant properties over time. This may involve the chemical stabilization of active compounds, as well as the refinement of processing and storage conditions. Addressing these challenges will be crucial to fully harness the potential of PLA/CNC nanocomposites for high-performance and sustainable applications.

## Figures and Tables

**Table 1:** PLA-based film formulations containing PEO, and pristine and irradiated CNC.

PLA-Based Sample	% PEO	% CNC	% Irradiated CNC
PLA/1PEO	1	-	-
PLA/1PEO/1CNC	1	1	-
PLA/1PEO/1CNC_25_	1	1	(25 kGy)
PLA/1PEO/1CNC_50_	1	1	(50 kGy)
PLA/2PEO	2	-	-
PLA/2PEO/2CNC	2	2	-
PLA/2PEO/2CNC_25_	2	2	(25 kGy)
PLA/2PEO/2CNC_50_	2	2	(50 kGy)

**Table 2:** Assignments of the main IR bands of PLA-based composites.

Bands	Wavenumber (cm^−1^)
–CH_2_ antisymmetric and symmetric stretching	2997–2945
–C=0 stretching	1747
–CH_3_ antisymmetric and symmetric bending	1300–1500
C–O–C stretching of esters	1266–1209
C–O–C stretching of PLA	1180
C–O stretching	1125, 1080, 1040
Amorphous and crystalline phases of PLA	868–755

**Table 3:** Measured parameters from DSC tests (second heating cycle) of neat PLA/PEO and PLA/PEO/CNC films.

	T_g_ (°C)	ΔH_c_ (J/(g·°C)	Tc Max (°C)	ΔH_m_ (J/g)	T_m_ Max (°C)	Xc (%)
PLA	61	35.30	112	43.70	167	9.03
PLA/1PEO	57	54.40	101	82.60	166	30.62
PLA/1PEO/1CNC	57	29.60	104	49.90	167	22.27
PLA/1PEO/1CNC_25_	57	31.10	104	51.10	166	21.94
PLA/1PEO/1CNC_50_	57	34.40	108	49.90	167	17.01
PLA/2PEO	55	21.40	113	56.60	183	38.62
PLA/2PEO/2CNC	56	18.80	97	56.40	167	42.11
PLA/2PEO/2CNC_25_	53	21.00	97	53.80	166	36.73
PLA/2PEO/2CNC_50_	56	11.20	98	43.70	166	36.40

**Table 4:** Measured parameters from mechanical tensile tests of neat PLA/PEO, and of composite films containing pristine and irradiated CNC.

	Stress at Break (MPa)	Elongation at Break (%)	Young Modulus (MPa)
PLA/1PEO	21.6 ± 2.4	1.0 ± 0.0	4387.0 ± 430.9
PLA/1PEO/1CNC	21.7 ± 1.6	1.3 ± 0.2	4994.4 ± 353.2
PLA/1PEO/1CNC_25_	14.7 ± 2.3	1.3 ± 0.2	4125.0 ± 279.6
PLA/1PEO/1CNC_50_	25.4 ± 2.5	2.2 ± 0.5	3769.6 ± 371.4
PLA/2PEO	19.7 ± 2.4	1.1 ± 0.1	4700.4 ± 285.4
PLA/2PEO/2CNC	17.7 ± 2.2	1.2 ± 0.1	4320.9 ± 257.6
PLA/2PEO/2CNC_25_	24.0 ± 1.6	2.1 ± 0.4	4385.9 ± 184.9
PLA/2PEO/2CNC_50_	20.0 ± 1.3	2.0 ± 0.5	4143.1 ± 260.2

**Table 5:** Water and diiodomethane contact angle data of PLA/PEO and of composite films containing pristine and irradiated CNC, as well as their surface free energy (γ_s_) and its dispersive (γ_s_^d^) and polar components (γ_s_^p^).

	Water Contact Angle (°)	Diiodiomethane Contact Angle (°)	$\gamma_{s}^{d}$ (mN/m)	$\gamma_{s}^{p}$ (mN/m)	$\gamma_{s}$
PLA/1PEO	87.4 ± 1.7	56.8 ± 1.7	30.42	2.97	33.38
PLA/1PEO/1CNC	88.8 ± 2.3	58.2 ± 1.8	29.61	2.71	32.32
PLA/1PEO/1CNC_25_	91.6 ± 1.3	52.7 ± 1.6	32.76	1.47	34.23
PLA/1PEO/1CNC_50_	89.9 ± 2.2	56.9 ± 1.4	30.36	2.26	32.62
PLA/2PEO	86.9 ± 3.2	61.6 ± 1.4	27.65	3.74	31.4
PLA/2PEO/2CNC	83.9 ± 1.0	55.7 ± 2.0	31.05	3.98	35.03
PLA/2PEO/2CNC_25_	90.9 ± 2.7	57.4 ± 1.4	30.07	2.05	32.12
PLA/2PEO/2CNC_50_	94.8 ± 2.8	61.4 ± 1.4	27.77	1.5	29.27

**Table 6:** Color analysis of PLA/PEO and of composite films containing pristine and irradiated CNCs.

	L*	a*	b*	Total Color (ΔE)
Reference	96.30 ± 0.09	0.50 ± 0.04	2.90 ± 0.14	0.00
PLA/1PEO	94.70 ± 0.06	0.50 ± 0.00	3.20 ± 0.16	1.63
PLA/1PEO/1CNC	95.30 ± 0.06	0.50 ± 0.06	2.90 ± 0.45	1.00
PLA/1PEO/1CNC_25_	94.10 ± 0.16	0.50 ± 0.06	4.70 ± 0.26	2.84
PLA/1PEO/1CNC_50_	94.5 ± 0.23	0.50 ± 0.06	3.7 ± 0.23	1.97
PLA/2PEO	95.00 ± 0.15	0.50 ± 0.00	3.1 ± 0.06	1.32
PLA/2PEO/2CNC	94.70 ± 0.06	0.40 ± 0.06	3.80 ± 0.26	1.84
PLA/2PEO/2CNC_25_	93.60 ± 0.06	0.30 ± 0.00	4.50 ± 0.35	3.14
PLA/2PEO/2CNC_50_	95.10 ± 0.21	0.50 ± 0.00	4.50 ± 0.67	2.00

## Data Availability

The original contributions presented in this study are included in the article. Further inquiries can be directed at the corresponding author.
